# 3D printed transtibial prosthetic sockets: A systematic review

**DOI:** 10.1371/journal.pone.0275161

**Published:** 2022-10-10

**Authors:** Sunjung Kim, Sai Yalla, Sagar Shetty, Noah J. Rosenblatt

**Affiliations:** 1 Dr. William M. Scholl College of Podiatric Medicine’s Center for Lower Extremity Ambulatory Research (CLEAR), Rosalind Franklin University of Medicine and Science, North Chicago, Illinois, United States of America; 2 Bionic Prosthetics & Orthotics, Merrillville, Indiana, United States of America; Semnan University, ISLAMIC REPUBLIC OF IRAN

## Abstract

The prosthetic socket, which transfers load from the residual limb to the prosthesis, is an integral part of the prosthesis. 3D printing has emerged as a potentially viable alternative to traditional fabrication for producing sockets that effectively transfer loads. We conducted a systematic review to better understand the current state of this newer fabrication method, with a focus on the structural integrity of 3D printed sockets and factors that can affect the strength of 3D printed sockets when tested using ISO 10328 standards. Literature searches were carried out in five databases (PubMed, Scopus, CINAHL, Web of Science and Google Scholar). Two reviewers independently performed the literature selection, quality assessment, and data extraction. A total of 1023 unique studies were screened in accordance with inclusion and exclusion criteria. Of 1023 studies, 12 studies met all inclusion criteria, with failure data for 15 3D-printed sockets and 26 standard laminated sockets. Within 3D printed sockets, the addition of composite materials such as carbon fiber particles and distal reinforcement using a compositing infill technique appears to improve socket strength. In light of the considerable amount of heterogeneity between studies in terms of materials and alignment used, the absolute values for failure could not be established for 3DS nor directly compared between 3DS and LCS. However, there is some evidence that the probability of a failure at a given load may be comparable between 3DS and LCS up to the P8 level. For all sockets, whether a laminated composite socket or a 3D printed socket, failure mainly occurred at the distal end of the socket or the pyramid attachment, which is consistent with the ISO testing protocol. Improving the strength of the 3D printed sockets through design modifications at the distal end and implementing emerging printing technologies could help to promote 3D printed sockets as a viable option, particularly when cost or access to care is limited.

## 1. Introduction

The prosthetic socket is an integral part of lower limb prostheses. Loads transfer from the residual limb to the prosthesis via the socket [1], which absorbs the impact forces felt on the residual limb [2, 3]. During normal gait the socket is subjected to repetitive loading with peak ground reaction forces reaching 110–120% of body weight [4]. Activities such as jumping, turning, and altering speed can lead to even greater, and more rapid loading [5]. To avoid mechanical failure, the ultimate force (failure force) of the prosthetic socket must be high enough to safely handle the daily accumulation of these stresses.

Thermoplastic and laminated composites are commonly used for fabricating traditional sockets [6] and advances in these materials provide for exceptional strength-to-weight characteristics. However, traditional methods for fabricating laminated composite sockets (LCS) require costly infrastructure, considerable input from prosthetists, and long production times, which limits rapid implementation of modifications to the final product [7, 8]. An alternative technique to fabricate prosthetic sockets is 3D printing, or additive manufacturing. The use of 3D printing in medical applications is rapidly growing and it has become a robust tool for fabricating complicated objects in a timely manner [9, 10]. Rapid production of 3D printed sockets (3DS) may allow for less time between amputation and receipt of the first prosthesis [11] or receipt of modified sockets following socket-related issues, which could collectively lead to better outcomes [12] and limit any negative effects of socket disuse [13]. Rapid production may also be relevant for managing patients with unstable limb volume who require many socket modifications. Moreover, the cost of a 3D printer and 3D printing filament materials is considerably lower than the costs associated with conventional manufacturing methods [14–16], making them a viable option for use in developing countries [17, 18].

Despite the potential benefits of using 3D printing technology in socket fabrication, the extent to which 3D-printing can provide sockets with ultimate forces that are appropriate for safe, long-term use is not entirely clear. Indeed, there are limited studies evaluating the use of 3DS in clinical practice, and those that do so have evaluated outcomes over fairly short time frames (2–6 weeks) [17, 19]. There is a lack of research on the ultimate force and durability of 3DS, potentially making prosthetists less confident in their use, despite considerable technological advancements, e.g., stronger filaments with high toughness and stiffness, that should improve safety.

The purpose of this study was to systematically review the existing literature regarding mechanical testing of 3D printed sockets in order to: 1) analyze factors that can affect the strength of 3D printed sockets; and 2) better understand the extent to which the structural integrity of 3DS is comparable to that of LCS.

## 2. Materials and method

### 2.1 Search strategy

Searches were conducted in the following databases: Medline (PubMed), Scopus, CINAHL, Web of Science. Key words related to strength, lower extremity, ISO10328, and their variations were combined for the search as shown in Table 1. Medical subject heading (MeSH) terms were used when appropriate. There was one additional record obtained from Google Scholar using a similar combination of keywords. The search produced a total of 1,505 hits, among which there were 482 duplicates, for a total of 1023 unique studies.

**Table 1:** Search strings used in each database.

Database	Search String
PubMed	(((((((((((((((strength) OR (static)) OR (failure)) OR (ultimate)) OR (load))) OR (loading)) OR (deformation)) OR (stress)) OR (strain)) OR (compressive)) OR (compression)) OR (ISO 10328)) OR (ISO 22523)) AND (socket)) AND ((("Tibia"[Mesh]) OR ("Artificial Limbs"[Mesh])) OR ("Lower Extremity"[Mesh]))
Scopus	( ( TITLE-ABS-KEY ( lower-extremity ) OR TITLE-ABS-KEY ( tibia ) OR TITLE-ABS-KEY ( artificial-limbs ) ) ) AND ( TITLE-ABS-KEY ( socket ) ) AND ( ( TITLE-ABS-KEY ( strength ) OR TITLE-ABS-KEY ( static ) OR TITLE-ABS-KEY ( failure ) OR TITLE-ABS-KEY ( ultimate ) OR TITLE-ABS-KEY ( load ) OR TITLE-ABS-KEY ( loading ) OR TITLE-ABS-KEY ( deformation ) OR TITLE-ABS-KEY ( stress ) OR TITLE-ABS-KEY ( strain ) OR TITLE-ABS-KEY ( compressive ) OR TITLE-ABS-KEY ( compression ) OR TITLE-ABS-KEY ( iso10328 ) OR TITLE-ABS-KEY ( iso22523 ) ) ) AND ( LIMIT-TO ( PUBSTAGE , "final" ) OR LIMIT-TO ( PUBSTAGE , "aip" ) ) AND ( LIMIT-TO ( SUBJAREA , "MEDI" ) OR LIMIT-TO ( SUBJAREA , "ENGI" ) ) AND ( LIMIT-TO ( LANGUAGE , "English" ) ) AND ( LIMIT-TO ( SRCTYPE , "j" ) )
CINAHL	(((((((((((((((strength) OR (static)) OR (failure)) OR (ultimate)) OR (load))) OR (loading)) OR (deformation)) OR (stress)) OR (strain)) OR (compressive)) OR (compression)) OR (ISO 10328)) OR (ISO 22523)) AND (socket)) AND ((("Tibia") OR ("Artificial Limbs")) OR ("Lower Extremity"))
Web of Science	TOPIC: (Strength* OR Static* OR Failure* OR Ultimate* OR Load* OR Loading* OR Deformation* OR Stress* OR Strain* OR Compression* OR ISO10328* OR ISO22523*) *AND* TOPIC: (Lower-Extremity* OR Tibia* OR Artificial-Limbs) *AND* TOPIC: (Socket*)
Google Scholar	(Strength OR Transtibial OR Prosthetic OR Socket) AND (ISO10328)

### 2.2 Inclusion and exclusion criteria

In order to be considered eligible for review, studies needed to include: 1) lower-limb prosthetic sockets; 2) mechanical testing for structural integrity performed according to ISO 10328 standards [20]. Studies were excluded if they: 1) did not include outcomes related to measures of socket strength, e.g., ultimate/failure force 2) were reviews or were not a full text article; 3) were published in a language other than English; and 4) included strength outcomes based on finite element analysis (FEA).

### 2.3 Selection process

Two blinded reviewers (SVY and SK) assessed the articles for initial inclusion and exclusion criteria. Studies with irrelevant titles or abstracts based on inclusion and exclusion were removed. Any conflicts between the two reviewers were resolved by a third reviewer (NJR).

### 2.4 Quality assessment of studies

The quality of all included studies was evaluated using a modified version of the standard National Institutes of Health (NIH) based quality assessment. Because the NIH-based quality assessment is focused on human subject trials, we modified the questions to focus on mechanical testing of sockets. We added 3 sub-questions (a-c) to the original questions 4 and 6, and 2 sub-questions to the original question 10. The final assessment tool (S1 Appendix) included a total of 16 items (questions and sub-questions), each scored 1 for yes or 0 for no/unclear. Each of the 16 items were ranked in order of importance with regard to impact on failure forces. For example, questions 4a and 4b were ranked as 1 and 2 respectively, as the topics they addressed (use of a settling test or a proof test) were expected to impact failure force. Similarly, question 11, which asked about presenting limitations and their impact on study outcomes, was given the lowest rank as it was not directly related to ultimate failure force. The rank order was reversed and then divided by 16 to calculate a weighting factor for each question (the top ranked item had a weight of 16/16 = 1 and the bottom ranked had a weight of 1/16). Scores on each of the 16 items were multiplied by their weighting factor and then summed for a mechanical-testing-specific quality assessment score that could range from 0–9.5. Quality assessment was performed independently by two authors (SVY and SK), and any disagreements on interpreting study quality were resolved by a third reviewer (NJR).

### 2.5 Quantitative analysis

We planned to limit our quantitative analysis to the socket orientation that was used most often. ISO 103280 standards describe socket orientation as a function of loading condition and load level (P-level). Importantly, socket orientation is identical for P5—P8 levels, with the differences only in settling and proof forces and the thresholds for safety [21]. Therefore, when choosing the orientation for quantitative analysis, tests performed at the P5 –P8 level were grouped together.

While it may be justifiable to simultaneously consider all sockets tested across three P-levels, in light of the numerous extraneous between-study differences that could impact strength (Table 2), the average force of 3DS and LCS across studies may poorly represent the strength of a generic socket. To overcome this, we considered two different, related approaches. First, using the raw data from all studies, we separately created cumulative distribution functions for failure force data for 3DS and LCS. Based on the extant literature, the cumulative distribution function at a given force value provides the probability of failure given that the failure load of a socket would be less than or equal to that force,. Typically, this function is analytically derived by calculating the cumulative integral of a distribution curve generated from a histogram of all data points. Considering the limited number of data points available, we simplified the approach and ordered force values from the lowest to highest, then assigned a cumulative probability to each value as 100 * (x/N), where *x* is the place in the order list and N is the total number of sockets. Each cumulative probability value was plotted at the corresponding force value. Comparing curves at a given force can help to draw a general conclusion that, in part, accounts for between-study differences; the probability can be thought to represent the likelihood of socket failure, assuming it was produced using a randomly chosen method among those currently in the literature for that socket type. Secondly, we expressed socket strength as a binary metric, i.e., whether or not the failure load surpassed the upper threshold for failure standards established by ISO 10328 at a given P-level. We then calculated the effect size for the difference in proportion of failures between 3DS and LCS by converting chi-squared values to Cohen’s d values [22]. Finally, we looked at the mode of failure for the different types of sockets.

**Table 2:** Summary of all studies evaluating ultimate strength of 3DS and/or LCS.

**Author**	**Year**	**Fabrication**	**Material Type**	**Matrix Type**	**Reinforcement Type**	**Extraneous Variable**	**Overall Sample Size**	**Multiple samples tested?**	Condition/P-level (P)^@^	**Failure Force**
Owen et al. [23]	2020	Traditional	PETG^#^	N/A		Manufacturers, limb type	5	YES	C2/P5	4091.78 ± 477.30
Pousett et al. [8]	2019		Orfitrans Stiff^#^			Cushion attachment, limb type	6	NO	C1/P3, P4, P5 C2/P3, P4, P5	11264, 11608, 12566
										4340, 4434, 5958
						Locking attachment, limb type	6			6091, 5241, 7650
										2763, 2853, 3000
Gerschutz et al. [6]	2012		PETG, Thermo-Lyn rigid, Orfitrans Stiff^#^			Manufacturers thickness limb type, pylon length	34	YES	C2/P6	2168 ± 1056.61
			Copolymer^#^				31			1181.87 ± 722.68
Current et al. [24]	1999		Composite	Acrylic Resin	Unidirectional carbon	limb type	2		C2/P5	3160 ± 155.56
					Carbon fiberglass stockinette		2			3073 ± 615.18
					Fiberglass stockinette		2			2409 ± 380.42
					Carbon cloth		2			2218 ± 73.53
					Fiberglass cloth		2			1836 ± 48.08
Owen et al. [23]	2020			Resin	Carbon fiber	Manufacturer, limb type	5		C2/P5	5575.4 ± 1039.73
							5			6462.26 ± 74.79
Graebner et al. [25]	2007			Foresee Epoxacryl		Type of lay-up, limb type	1	NO	C2/P5	5663
							1			5380
							1			5494
							1			5434
							1			4247
Campbell A et al. [26]	2012			Acrylic Resin	Nyglass stockinette	limb type	1			5808
				Plant Oil Resin			1			4255
**Author**	**Year**	**Fabrication**	**Material Type**	**Matrix Type**	**Reinforcement Type**	**Extraneous Variable** ^**^**^	**Overall Sample Size**	**Multiple samples tested?** ^**&**^	**Condition (C)/ P-level (P)** ^**@**^	**Failure Force** *
Pousett et al. [8]	2019	Traditional	Composite	Resin	Nyglass & carbon cloth	Cushion attachment, limb type	1	NO	C1/P3, P4, P5 C2/P3, P4, P5	13341, 12113, 13132
										4384, 4384, 6505
						Locking attachment, limb type	1			10058, 10364,11730
										2818, 3278, 3526
Graebner et al. [25]	2007			Foresee Epoxacryl	Nyglass	Type of lay-up, limb type	1		C2/P5	5325
							1			5325
							1			3841
							1			4235
							1			2726
Campbell A et al. [26]	2012			Plant Oil Resin	Ramie	limb type	1			6180
				Acrylic Resin			1			4657
Neo et al. [27]	2001			(Not Specified)	(Not Specified)	limb type	1		C1/P5	No failure
									C2/P5	
Gerschutz et al. [6]	2012			Resin	Carbon fiber & fiber glass	Manufacturers, thickness, limb type	33	YES	C2/P6	4273.33 ± 1049.09
Türk et al. [28]	2018	3D printed	Carbon fiber (CF)	N/A		limb type	1	NO	C2/P5^!^	7685
Nickel et al. [21]	2020					limb type	5	YES	C2/P6	4862 ± 191
Stenvall et al. [29]	2020		Polypropylene (PP)			Force orientation, limb type	1	NO	C2/P6	2836
Goh et al. [30]	2002					Manufacturer	1		C2/P5	4025
Pousett et al. [8]	2019		Polylactic Acid (PLA)			Cushion attachment, limb type	6		C1/P3, P4, P5 C2/P3, P4, P5	10925, 9197, 9355
										2020, 2189, 2243
						Locking attachment, limb type	6			5107, 7001, 6725
										4355, 4707, 4133
Campbell et al. [31]	2018					Infill 30%	3	YES	C1/P5	10018 ± 4064.30
						Infill 40%	3			10245 ± 1190.11
						Infill 50%	3			11351± 1310.47
Owen et al. [23]	2020					Manufacturer, limb type	5		C2/P5	3836.85 ± 478.37

* For studies that used more than one sample, failure forces are presented as mean+/-SD

@ refers to the targeted load/condition at the failure point

^ extraneous variables are defined as dependent variables that have the potential to affect the results

& refers to multiple samples tested at a given condition and load level; studies may have a sample size of > 1 but not “multiple samples tested” if only one sample was tested at each condition and P-level combination

# refers to thermoplastic materials, which are not analyzed in the main text but included for completeness

! author set a new target load by multiplying the P5 load level by a safety factor of 36%.

## 3. Results

### 3.1 Study selection

**Fig 1:**
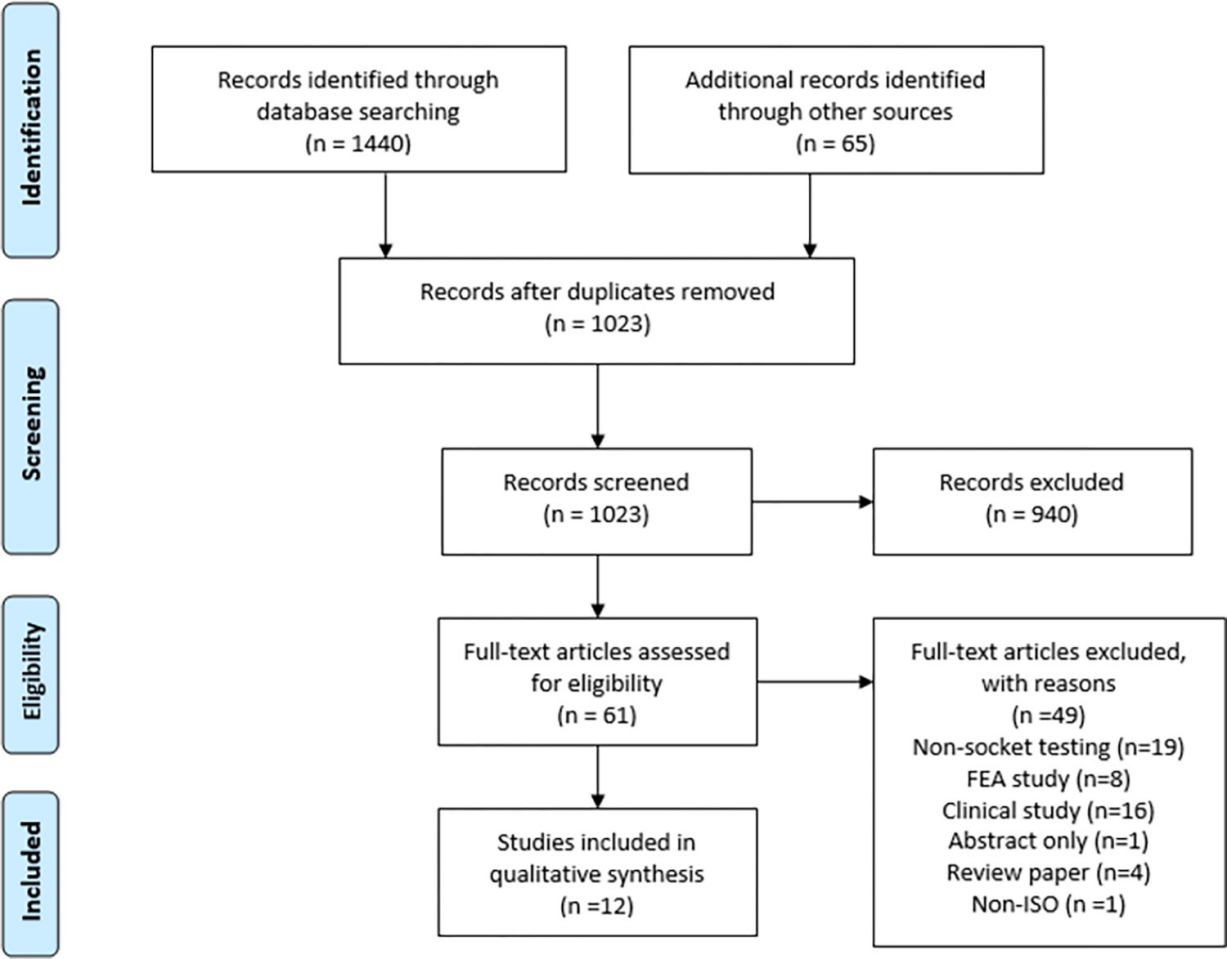
Flow chart of the literature search and study selection.

The screening and study selection were reported following the Preferred Reporting Items for Systematic Review and Meta-Analyses (PRISMA) statement (Fig 1). Of the 1023 unique studies considered, 940 studies were excluded based on eligibility criteria. Thereafter, 61 full-text articles were assessed for eligibility, of which 10 were initially included based on agreement between the two reviewers. Among the 61 full-text articles assessed for eligibility, 49 articles were excluded due to absence of socket testing, use of FEA, being a prospective or observational clinical study, or for being a systematic review/abstract. Fourteen conflicts between the two reviewers were resolved by a third reviewer (NJR), based on which two additional studies were included. In total, 12 studies were included for our assessment.

### 3.2 Quality assessment of studies

Quality assessment score for the 12 included studies ranged from 4.44–9.06 (maximum possible of 9.5) with an average score of 6.68 ±1.38 (Table 3). Studies with the highest quality scores followed ISO 10328 guidelines, examined different levels of exposures, and clearly reported outcome measures. Most of the studies failed to provide sample size justification, partly reflecting the use of a single sample, which precludes statistical analyses. The largest between-study variance in quality scores was attributable to question 4, where many studies received no points as they did not include a settling or proof test when performing the ultimate failure test. The study that received the lowest score focused more on 3D printing techniques rather than providing details of testing methods or procedures.

**Table 3:** Quality assessment within included studies. For description of score items, see S1 Appendix.

**Lead**	**Years**	**Q1**	**Q2**	**Q3**	**Q4a**	**Q4b**	**Q4c**	**Q5**	**Q6a**	**Q6b**	**Q6c**
**authors**											
Pousett	2019	0.5	0.5625	0.3125	1	0.9375	0.8125	0	0.875	0.75	0.6875
Campbell	2018	0.5	0.5625	0.3125	1	0.9375	0.8125	0	0.875	0.75	0.6875
Nickel	2020	0.5	0.5625	0.3125	0	0.9375	0.8125	0.4375	0.875	0.75	0.6875
Current	1999	0.5	0.5625	0.3125	1	0	0.8125	0	0.875	0.75	0.6875
Owen	2020	0.5	0.5625	0.3125	0	0.9375	0.8125	0	0.875	0.75	0.6875
Gerschutz	2012	0.5	0.5625	0.3125	0	0	0.8125	0.4375	0.875	0.75	0.6875
Graebner	2007	0.5	0.5625	0.3125	0	0	0.8125	0	0.875	0.75	0.6875
Campbell. A	2012	0.5	0.5625	0	1	0	0.8125	0	0.875	0	0.6875
Goh	2002	0.5	0.5625	0.3125	0	0.9375	0.8125	0	0.875	0.75	0.6875
Neo	2000	0.5	0.5625	0.3125	1	0	0.8125	0	0.875	0.75	0
Stenvall	2020	0.5	0.5625	0.3125	0	0	0.8125	0	0.875	0.75	0
Türk	2018	0.5	0.5625	0.3125	0	0	0.8125	0	0.875	0	0.6875

		**Q7**	**Q8**	**Q9 **	**Q10a**	**Q10b**	**Q11**	**Sum**	**Rank**
Pousett	2019	0.625	0.4375	0.4375	0.4375	0.4375	0.25	9.06	1
Campbell	2018	0.625	0.4375	0.4375	0	0	0.25	8.19	2
Nickel	2020	0.625	0.4375	0.4375	0	0.4375	0.25	8.06	3
Current	1999	0	0.4375	0.4375	0.4375	0.4375	0.25	7.50	4
Owen	2020	0.625	0.4375	0.4375	0	0	0.25	7.19	5
Gerschutz	2012	0	0.4375	0.4375	0.4375	0.4375	0.25	6.94	6
Graebner	2007	0.625	0.4375	0.4375	0	0	0.25	6.25	7
Campbell. A	2012	0.625	0.4375	0.4375	0	0	0.25	6.19	8
Goh	2002	0.625	0	0	0	0	0	6.06	9
Neo	2000	0.625	0	0	0	0	0	5.44	10
Stenvall	2020	0	0.4375	0.4375	0	0	0.25	4.94	11
Türk	2018	0	0	0.4375	0	0	0.25	4.44	12

### 3.3 Features impacting socket strength

The following information was extracted from all of the included studies: socket fabrication method (e.g., 3D printed vs traditional which included LCS and check sockets) and type of material; loading alignment and level for the socket test; sample size (number of sockets tested at each loading condition and P-level); failure force; extraneous variables other than the ones mentioned above that could impact failure force within a study and contribute to heterogeneity (e.g. manufacturer, limb shape, number of layups) (Table 2).

#### 3.3.1 Socket fabrication and materials

Five studies [21, 28–31] used only 3D printing techniques for socket fabrication; five studies [6, 24–27] used only traditional manufacturing techniques; and two studies [8, 23] used both additives and traditional manufacturing techniques. Among the studies using 3D printing techniques, one study [28] used multiple manufacturing techniques, i.e., selective laser melting (SLM), selective laser sintering (SLS), and fused deposition modelling (FDM), and the other six studies [8, 21, 29–31] used only FDM. Among the traditional manufacturing techniques, seven studies [6, 8, 23–27] used lamination for definitive sockets.

The materials commonly used in the 3D printing techniques were polylactic acid (PLA), polypropylene (PP), and carbon fiber (CF), or more precisely filaments of PLA reinforced with particles of carbon fiber that provide extra strength. For CF, the particles of carbon fiber in the final socket are not interwoven as they are in LCS reinforced with carbon fiber, hence they are not as strong. Three studies [8, 23, 31] used PLA, two studies [29, 30] used PP, and two studies [21, 28] used CF, while most LCS were made of carbon fiber and laminated with either nyglass or fiber glass [6, 8, 23–25].

#### 3.3.2 Loading alignment and level

**Fig 2:**
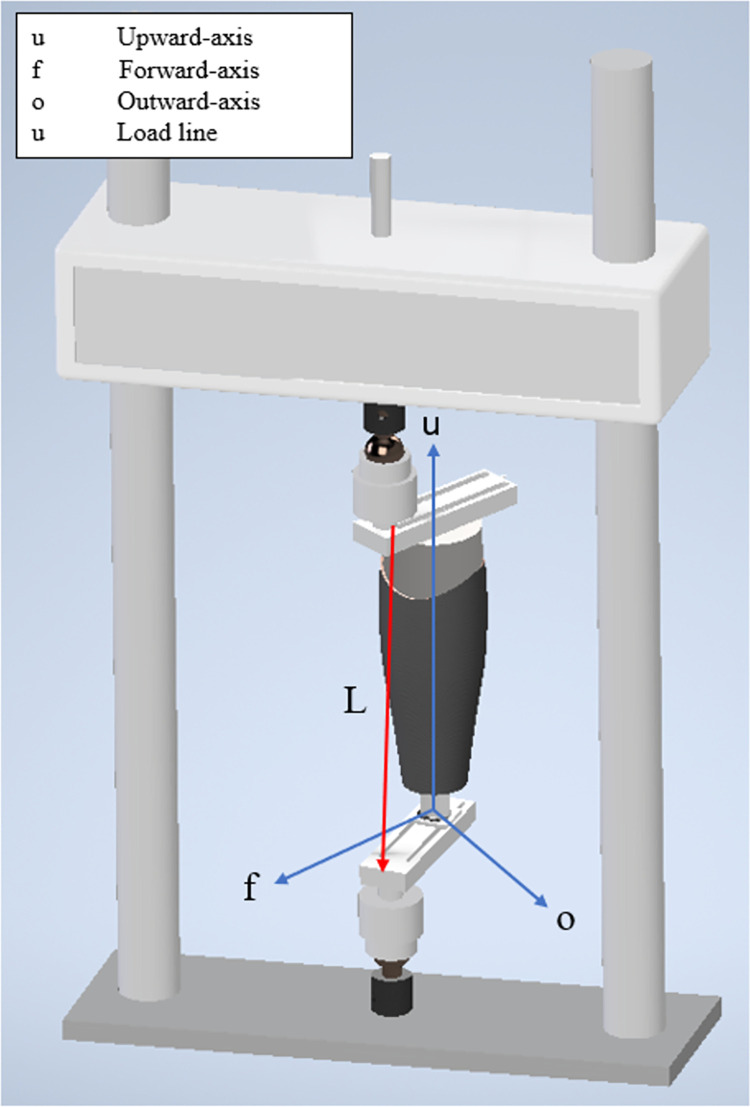
The socket alignment for Condition II and P-levels of P5 and above as described in ISO 10328. The proximal and distal adaptors are perpendicular to the u-axis. The shorter pylon is employed in this figure for simple visualization and does not reflect the ISO 10328 standard.

ISO 103280 standards describe loading that can occur at Condition I (which simulates toe off) or Condition II (which simulates heel strike) as well as the socket orientation (relative to load line) for these conditions (Fig 2), which varies as a function of load level (referred to as P-level in ISO 10328). Note that load level does not correspond to the amount of load applied to the socket during mechanical testing, but rather defines the level of force for the settling and proof test thresholds above which the socket is determined safe for use on a patient of a given body mass. For example, when performing testing at the P5-load level (used for assessing safety for a 100 kg patient), settling and proof tests occur at 920 N and 2013 N, respectively, and the socket is determined to pass the P5-level, i.e., is safe for use by a 100 kg patient, if the ultimate force surpasses 4025 N (twice the proof force) [20]. Condition I alignment was employed by 25% of studies [8, 23, 31] while 92% of studies performed testing under ISO Condition II [8, 21, 23–30]. Among all the studies, only one study used testing below the P5-level and tested one socket at each of the P3- and P4-levels. All twelve studies tested sockets at the P5-level and above (12 studies used P5, 2 studies used P6, 1 study used P7, and none of the studies used P8).

It is important to note that even at the same P-level, the socket alignment may vary depending on the relative orientation of the ankle to the knee joint. Indeed, the study of Gariboldi et al. identified three different types of test setup approaches under ISO standards [32], which were referred to as Neo [27], Current [24], and Gerschutz [6] alignment. Each relative orientation generated a different load line due to the different fixations of the ankle and knee joints as well as the length of the pylon. In this review, eight studies used the current alignment [8, 21, 23–26, 28, 31], two studies used the Neo alignment [27, 30], and two studies used the Gerchutz alignment [6, 29].

#### 3.3.3 Other extraneous variables

Multiple methodological differences (extraneous variables listed in Table 2) exist between studies and are relevant to consider when comparing socket strength across studies. Differences in manufacturing processes between fabrication facilities can impact socket shape and, in turn, strength. The study of Gerschutz et al. [6] had nine different facilities, each supplying four check sockets and four LCS for strength testing. Each facility was sent the same STL file along with a set of fabrication instructions. They reported that sockets provided by different facilities had different thicknesses of the distal curvature, resulting in variations in yield strength. With regard to the manufacturing of 3DS, the study by Nickel et al. [21] demonstrated that a patented compositing infill technique could increase the strength of the socket. Specifically, a gap was left in the socket during initial printing, which was then filled with carbon fiber inserts, similar to using rebar in concrete, but in this case to reinforce the u-axis (Fig 2) against delamination.

Additional methodological differences, including different limb models and pylon lengths, may also be relevant when comparing socket strength across studies. For example, the limb model of Gerschutz et al. [6] was an enormous square bucket, representative of an obese, likely sedentary, individual. The limb model of Owen et al. [23] was smaller in size and had a cylindrical or conical shape with no abnormal anatomy or bony protrusions, whereas the limb of Nickel et al. [21] was moderate in size and had a rather bulbous shape at the distal end with some bony protrusion. Different limb shapes can alter the magnitude of hoop and longitudinal stress acting on the inside of the socket wall. With regard to pylon length, the dimension established in the ISO standards is not concrete but rather is datum dimensioned, with all measurements starting from a common reference plane; when one part is lengthened, the other parts connected to it must be relatively shortened, so that the total dimension between reference-to-reference points, as defined by the ISO standards, is maintained. Campbell et al. [26] used a 27 cm long pylon but a thin adapter plate (2.5 cm long) below the ankle. The pylon length in Current et al. was a 23 cm long pylon with a 6 cm thick adapter plate below the ankle; the pylon length of Gerschutz et al. [6] was very short (5 cm long). Pylon length can change the failure location because the center of the socket changes depending on pylon length.

### 3.4 Quantitative analysis

#### 3.4.1 Strength comparison of 3DS and LCS

**Fig 3:**
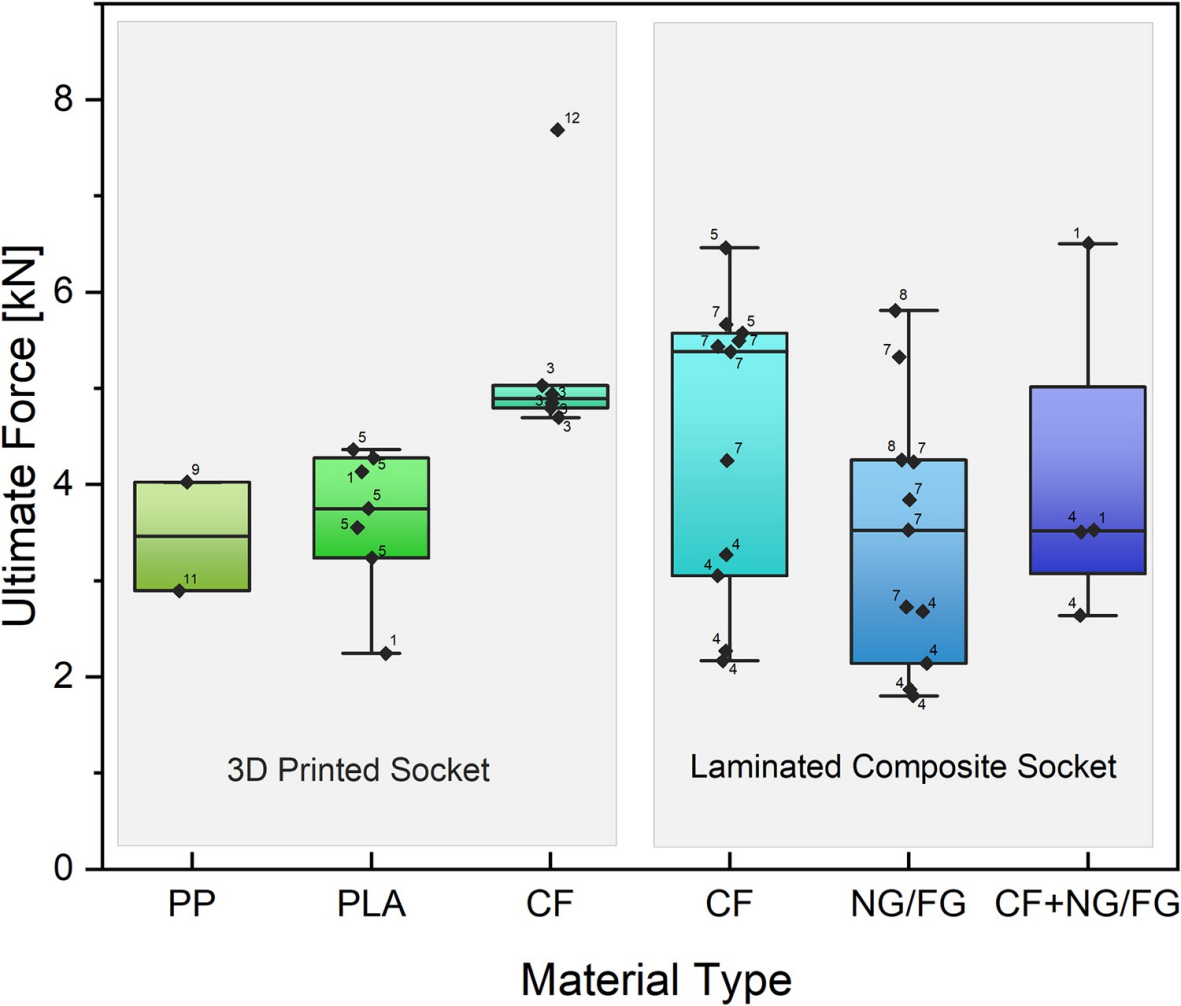
Bar and whisker plots for the ultimate force for six different materials under Condition II and P5-P level loading. The 3D printed socket materials include: polypropylene (PP), polylactic acid (PLA), and carbon fiber reinforced PLA (CF). For laminated composite sockets, material type refers to the reinforcement fiber, which includes: carbon (CF), nyglass/fiberglass (NG/FG), and carbon fiber with nyglass/fiberglass (CF + NG/FG). Each data point represents results from testing of a single sample. The number next to each data point indicates the rank of each study based on quality assessment, with 1 being the highest and 12 the lowest. The top and bottom of the box represent the first and third quartiles, with the median shown as a horizontal line inside the box. The whiskers show the 1.5 interquartile lower/higher range.

Although a number of between-study differences have been noted, we calculated average strength values for a very general sense of the strength of 3DS and the between-study variance therein. The average ultimate failure force for 3DS at P5-P8 level was 4298 ± 1236 N (Fig 3) which is within the ISO standards for P5 load level (3360–4480). Quality scores for each failure load have been incorporated into Fig 3, from which it can be seen that studies using 3DS made of CF had, on average, the highest quality (although the lowest quality study was also found here and accounted for the strongest 3DS reported). For reference, the average failure for LCS was 3946 ± 1490 N. The higher average ultimate force value for 3DS was, in part, driven by the one low quality data point for CF, whereas the average for LCS was in part driven down by high quality (rank 4) data points of relatively low failure for LCS with NG/FG only. Thus, in light of the number of between-study differences, these average values should not be thought to reflect the value one might expect, on average, if they were to produce a 3DS or LCS.

**Fig 4:**
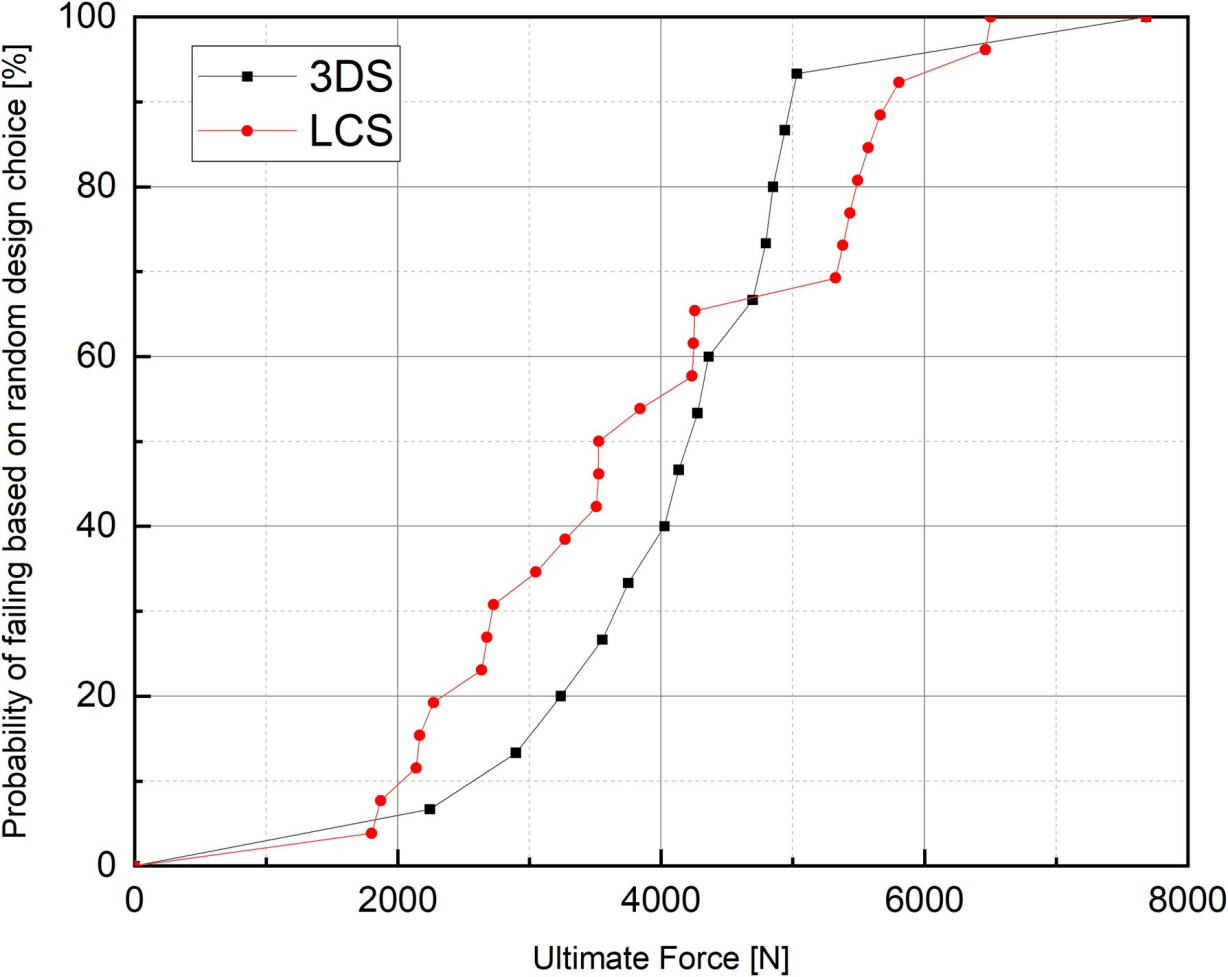
The probability of socket failure. The cumulative distribution function, which shows the probability of socket failure, implicitly accounts for methodological differences that exist between studies. The probability of socket failure for 3DS is similar to LCS except at high loads.

To better understand the available data on 3DS in light of what is known for LCS, while considering the large degree of heterogeneity, we created cumulative distribution functions (Fig 4). With this method, the probability of socket failure was somewhat higher for LCS at low loads, in part reflecting the fact that many more LCS were tested at P5 –P8 compared to 3DS. This does not exclude the likely scenario that any one combination of LCS fabrication methods would produce a socket that is stronger than most 3DS. Rather, the analysis accounts for this possibility while also considering the possibility that certain methods of LCS fabrication could result in sockets that are weaker than the majority of 3DS presented in the literature. At higher loads (>4500 N), the reverse trend seems to emerge, whereby the probability of failure at a given load is higher for 3DS than for LCS. As expected, this agrees with the binary outcome analysis (Table 4). The difference in proportion of failures corresponded to a very small effect at P5 -P7 load levels, while the effect was of moderate size at the P8-load level.

**Table 4:** The proportion of failure rate for 3DS and LCS.

P-level	Socket Type	Pass	Fail	Effect Size
P5	3DS	10	5	Small effect
	LCS	12	14	(d = 0.404)
P6	3DS	6	9	No effect
	LCS	9	17	(d = 0.107)
P7	3DS	4	11	Small effect
	LCS	9	17	(d = 0.205)
P8	3DS	1	14	Intermediate Effect
	LCS	9	17	(d = 0.660)

#### 3.4.2 Socket failure modes

For all sockets, whether LCS or 3DS, failure mainly occurs at the distal end of the socket or the pyramid attachment. Four studies [8, 21, 28, 29] reported that the 3DS broke circumferentially with cracks initiating near the pin lock hole and propagating from the lateral to the medial side. In the study by Owen et al. [23], medial cracking was also observed in 3DS, but four sockets failed at the pyramid attachment; the pyramid was either pulled out of the socket or broken.

The failure mode in the LCS was found more in the pyramid attachment than at the distal end of the socket [8, 24, 25] (Table 5). Current et al. [24] reported that six LCS had a complete failure at the pyramid attachment plate, resulting in the anterior edge of the pyramid attachment being pushed completely through the distal end of the sockets. Similarly, Graebner et al. [25] and Pousett et al. [8] observed that the pyramid was disconnected from the distal end of the socket. Failure at the distal end was also commonly found in LCS. Owen et al. reported that LCS failed at the anterior distal wall. In addition, several authors reported that pylon bending was observed in conjunction with socket failure for the LCS [8, 23, 30].

**Table 5:** Failure mode for 3DS and LCS after mechanical testing under Condition II and P5 loading level.

Failure mode	3DS	LCS
Medial Crack	10%	3.8%
Distal End Crack	50%	11.5%
Pyramid Disconnection	40%	65.3%
Pylon bending	0	11.5%

## 4. Discussion

We aimed to review the literature that tested the strength of lower extremity prosthetic sockets using the ISO 10328 standard in order to better understand how practices with 3DS impact strength, and the strength of 3DS relative to LCS. Although a recent review evaluated mechanical testing of prosthetic sockets [32], and 11 of the 16 articles therein are considered here, the prior review focused on strength testing methodology rather than on 3DS technology. A primary conclusion of that study was that ISO 10328 test standards are a reasonable reference for defining socket strength testing guidelines, which justifies our exclusion of studies that did not employ such a standard. The prior review also recommended that strength testing studies utilize more standardized protocols, including standardized socket and limb dimensions, and knee joint center orientation, to facilitate between-study comparisons [32]. Indeed, these between-study differences precluded any true meta-analytical approaches in our current review.

In our review, we observed a wide variety of materials used for 3DS, with little justification for material choice. Traditionally, PLA has been used in 3D printing of orthopedic devices when biosafety was being considered. Indeed, PLA is biocompatible and is not metabolically harmful [33, 34]. In addition, compared to acrylonitrile butadiene styrene (ABS), which is another common 3D printing material in FDM techniques, PLA has a lower melting point (150–162°C) [35, 36], higher compressive strength (70 MPa) [37], and lower thermal expansion (78 μm/m-K) [38] due to the semi-crystalline nature of PLA. Accordingly, PLA could have certain advantages for prosthetic sockets, such as higher durability and strength. Another material traditionally used for 3D printed devices is PP, which tends to be chosen due to its lower cost and better durability than other common 3D printing materials, e.g., ABS or PLA. However, only two of the studies reviewed [29, 30] used PP, and any strength benefits for 3DS are not definitive. Within the selected 3D printed studies, CF was commonly used, likely reflecting the fact that CF is widely known to have exceptionally high strength and be lightweight. Composite 3DS, e.g., adding carbon fiber particles to PLA resulted in a 31.5% increase in ultimate failure force when compared to PLA alone (Fig 3). Adding carbon to PLA has also been shown to improve bending modulus and maximum the bending strength of material samples by about 208% and 36%, respectively, relative to PLA alone [39]. These properties are important for socket strength testing since, under Condition II (the most commonly tested condition), the load line is anterior to the knee and produces a bending moment in the socket (Fig 2). The higher strength of carbon fiber reinforced PLA, in part, may contribute to the generally similar probabilities of failure for 3DS and LCS at P5-P7 load levels.

Of course, carbon fiber is also one of the most commonly used materials for reinforcing LCS, and reinforcing LCS with CF, may provide greater strength to LCS compared to NF/FG alone (Fig 3). CF reinforced LCS likely contributes to an apparent reduced failure probability of LCS versus 3DS at high loads (Fig 4). Reinforced LCS may, in part, be stronger than 3DS as the fabrication method allows for greater fiber volume fraction, e.g., increasing lay-up number, with fibers that can run in many different directions. Overall, the higher volume fraction results in better mechanical performance of the LCS.

While there is evidence that the probability of 3DS failure may be similar to, or even lower than, that of LCS at the lower loading level, some differences may exist with increasing load levels. Whereas the proportion analysis showed minimum effects of fabrication type on failure rates between 3DS and LCS at the P5-P7 level, the effect may be moderately strong at the P-8 level, whereby 3DS may be weaker and more likely to fail (Table 4). Due to the inherent nature of the layer-by-layer structure, 3DS are generally susceptible to shear failure, and the likelihood of this failure mode may increase with increasing load. Improving the strength of 3DS at the P8 loading level should be a focus of future research and is particularly relevant in light of the relationship between obesity and amputation [40, 41]. The extant literature provides some insight into the possible means to do so. Nickel et al. [21] showed that improving the design process with iterative structural testing can improve the overall strength of sockets. Annealing can also help in reinforcing 3D printed materials as per manufacturing recommendation [42], as can the addition of fibers around the distal end of the socket [24]. Such modifications to the socket and socket design process, when combined with technological advancements in 3D printing filaments (e.g., the addition of carbon), may lead to 3D printed composite sockets that are viable for P8 load levels.

Importantly, consideration of ultimate failure alone to assess socket viability and safety may be overly conservative. For example, although Pousett et al. demonstrated that LCS had a considerably higher ultimate force compared to 3DS, they found that the ultimate force of 3DS (printed with PLA alone) (4707 N) sufficiently passed the ISO standards for both the lower and upper thresholds of the P5 load level (4025 N), without a catastrophic failure. This suggests the 3DS is able to permit plastic deformation by absorbing energy safely beyond ISO thresholds. With regard to the safety of 3DS for obese individuals, even a 175 kg person may only experience forces of around 2000N during gait (e.g., 110–120% body weight) [43], and the ultimate force for 3DS at the P8 level is high enough to withstand these forces. In addition, the ultimate force of 3DS during daily use is likely to be higher than that seen during isolated socket testing given that strength testing fails to consider the impact of using prosthetic feet that have energy-storing properties [44], which is likely to increase the failure loads of the full prosthetic set-up relative to the socket alone.

Based on current data on mechanical testing using ISO standards, one may assume that regardless of fabrication method or material, the socket body and proximal extensions are unlikely to fail. Indeed, the failure of sockets often appeared on the pyramid attachment plate and distal end regardless of the fabrication method (Table 5). Several studies reported large bending moments at the distal end of the socket when tested at Condition II, causing the anterior edge of the pyramid attachment plate to act as a focal point for a stress riser [24, 25]. Distal failures might be due to the holes in the lamination for the pin lock mechanism that may promote crack initiation [24, 25]. However, the extent to which the socket is susceptible to failure at more proximal locations is not clear as the ISO standards, by design, promote stress concentration at the distal end of the socket to primarily assess the strength of the connection between the socket and componentry rather than the componentry itself. In fact, in a recent clinical trial of 3DS that allowed patients a 2-week accommodation period with a 3DS, one participant experienced socket failure at the brim region of the socket during accommodation [19]. Perhaps this reflects the fact that areas proximal to the distal end of the socket, e.g., body prominences such as the fibular head, experience higher peak pressure throughout the stance phase than do more distal locations [45]. Given that current testing standards are optimized to assess the interaction between distal end and connector (such as pyramid adaptors), and concentrated forces focus on the distal end, it is not surprising then that ultimate force improved in LCS by multiple layups on the distal end of the socket [25]. While the testing standard does not provide adequate loading to the central and proximal regions of the socket, the study from Owen et al. [23] reported failure at regions proximal to the distal end, indicating that, to some extent, ISO testing standards may still partly load other regions of the socket.

Failure modes that do not relate to damage at the distal end or pyramid attachment may be relevant to 3DS failure. For example, shear failure may be a common occurrence in 3DS due to greater shear forces between layers [8, 31]. Fortunately, shear failure can be reduced by modulating different printing parameters, such as raster angle, infill density, infill pattern, and adding corrugations [46–48]. Furthermore, new 3D printing techniques such as solid pass with high flow no longer require an inner and outer wall, eliminating the need to consider infill percentage. Future work should focus on exploring the extent to which socket strength can be improved by using these techniques as well as by providing a stronger layer around the pin holes and/or changing the design at the distal end to have a more robust attachment to the pyramid and pylon.

Despite seemingly similar probabilities of failure for 3DS and LCS, at least at P5-P7 loads, and despite the great potential for 3DS in terms of reaching underserved regions [17] or managing patients with challenges related to limb volume, 3DS has not yet been adopted for daily clinical use. The lack of wide scale adoption may, in part, reflect safety and durability concerns by prosthetists as well as concerns related to 3DS socket comfort [12]. Nonetheless, preliminary experimental clinical data suggests that 3DS may be safe and effective for daily use. For example, a clinical trial in Sierra Leone that included eight participants reported no failures in 3DS over a 6-week trial period [17]. In addition, a recent study by Nickel at el. reported that performance-based outcomes were no worse after 2 weeks of using a 3DS and that users were accepting of 3DS [19]. Nonetheless, there is still a significant lack of evidence regarding the durability (fatigue testing) of 3DS (Table 6). As seen from Table 6, limited evidence exists on fatigue testing in laboratory conditions. Additional work is needed to not only demonstrate the durability of 3DS but also to understand factors that will increase the clinical utilization of 3DS.

**Table 6:** Summary of all studies comparing the fatigue strength of 3DS versus LCS.

Author	Year	Fabrication	Material Type	Matrix Type	Reinforcement Type	Extraneous Variable^^^	Overall Sample Size	Multiple samples tested? ^&^	Condition (C)/ P-load level(P)	Max. load (N)	Min. Load (N)	Frequency (Hz)	Number of cycles
Goh et al. [30]	2002	3D printed	Polypropylene (PP)	N/A		Manufacturer	1	NO	C1/P5 C2/P5	1330 1200	50	2	250,000
Türk et al. [28]	2018		Composite	Polymer (PLA, ABS, PETG etc.)	Carbon fiber	Hybrid Fabrication	1	NO	C2/P5	1614	50	6	3,000,000
Nickel et al. [21]	2020					Iterative Design Change	5	YES	C2/P6	1450	50	0.7	3,000,000
Neo et al. [27]	2001	Traditional		(Not Specified)	(Not Specified)	(Not Specified)	1	NO	C1/P5 C2/P5	1330 1200	50	3	3,000,000

^ extraneous variables are defined as dependent variables that have the potential to affect the results

& refers to multiple samples tested at a given condition and load level; studies may have a sample size of > 1 but not “multiple samples tested” if only one sample was tested at each condition and P-level combination.

One limitation of this study is the presence of a large number of diverse extraneous variables that can impact socket strength, which precludes any meta-analytic approaches. As already noted, another limitation is that the ISO 10328 standard primarily focuses on the loading at the distal end and it does not effectively assess the strength in other regions of the socket. However, no widely accepted guidelines exist on socket strength testing to focus on other regions of the socket, and many studies and clinicians use ISO 10328 as a standard for their socket testing. Future work is needed to standardize how the current ISO recommendations are applied and, more importantly, to develop new testing standards that do not specifically focus on the distal end of the socket. In the absence of a new standard, ISO 103280 remains our best tool.

## 5. Conclusion

The findings of our systematic review on the structural integrity of prosthetic sockets showed that there are a number of factors, for example socket material, testing alignment, or limb shape, that can impact the failure strength of 3DS. As a result of the large degree of heterogeneity that exists between studies, it is not possible to definitively state the ultimate failure of a given 3DS or to statistically compare the strength of a 3DS to LCS. Nonetheless, our review provides insight into 3D printing of sockets to inform future design, which includes:

1. The strength of 3DS made of PLA or PP filaments was lower than that for LCS in general, but it was enhanced by using a composite filament, such as the addition of carbon fiber particles;
2. The strength of 3DS can be improved by reinforcing the distal end with compositing infill techniques. We also suggest that new advances in printing technologies that include annealing of materials and alternating fiber directions may further enhance strength;
3. The probability of a randomly fabricated 3DS and LCS failing at a load below the P8 threshold may be comparable;
4. Failure modes of 3DS, as well as LCS, mainly appeared near the distal end, or socket adapter, which may be expected based on the ISO standards that focus on this region of the socket.

Overall, the strength of 3DS under the limited testing conditions shows promise to be used clinically for definitive sockets, especially for sockets designed for the P5 loading and when cost is an issue or access to care is limited.

## Supporting information

S1 Checklist PRISMA 2020 for abstracts checklist. (DOCX)

S2 Checklist PRISMA 2020 checklist. (DOCX)

S1 Appendix Quality assessment criteria. (DOCX)

## Abbreviations

LCS: Laminated Composite Socket
3DS: 3D printed Socke
FEA: Finite Element Analysis
NIH: National Institute of Health
PRISMA: Preferred Reporting Items for Systematic review and Meta-Analyses
SLM: Selective Laser Melting
SLS: Selective Laser Sintering
FDM: Fused Deposition Modelling
PLA: Polylactic Acid
PP: Polypropylene
CF: Carbon Fiber
PETG: Polyethylene Terephthalate Glyco
NG: Nyglass
FG: Fiberglass
