# Supplementary material for: 3D printed transtibial prosthetic sockets: A systematic review
Source: PLoS One. 2022 Oct 10;17(10):e0275161. doi: 10.1371/journal.pone.0275161 (PMC9550041; doi:10.1371/journal.pone.0275161)
Supplement: S1 Appendix — (DOCX) [file pone.0275161.s003.docx]

**S1 Appendix. Quality Assessment Criteria**

Q1: Was the research question or objective in this paper clearly stated?

Q2: Were criteria for the selection of sockets and type of mechanical test clearly described?

Q3: Were the type of sockets in the study representative of those who would be eligible for the

test in the general or clinical population of interest?

Q4: Were inclusion and exclusion (proof test etc.,) criteria for being in the study adequately described and applied uniformly to all sockets? (Were all specimens included in the analysis?)

Static tests

(a) Was settling test performed for the each of the socket before failure loading?

(b) Was proof test performed for the each of the socket before failure loading?

(c) Was each of the socket tested only once (not reused for testing)?

Q5: Was a sample size justification or variance sufficiently provided?

Q6: Was the test clearly described (use of standards, testing condition, socket dimensions etc.,) and delivered consistently across the sockets?

Static tests

(a) Were the tests performed according to ISO 10328 standard?

(b) Clear concise description of testing method such as loading level, condition reported?

(c) Was specific dimension, thickness of walls, and weight of the sockets reported

Q7: For material testing that can vary in amount or level, did the study examine different levels of the exposure (settling test, proof test, different conditions tested) as related to the outcome (e.g., categories of exposure, or exposure measured as continuous variable)?

Q8: Were the outcome measures (dependent variables - force, deformation) clearly defined, valid, reliable, and implemented consistently across sockets?

Q9: Were the exposure measures (independent variables - thickness of socket, fiber direction or infill percentage) clearly defined, valid, reliable, and implemented consistently across all study?

Q10: Were statistical tests done appropriate that provided type of model and p values for the pre-to-post changes?

(a) The selected statistical model is adequate for the design of the study

(b) Provided or mentioned p-value in analysis result

Q11: Were key potential confounding variables (potential limitations) measured and explained for their impact on outcome(s)?
